# *Wuchereria bancrofti* infection at four primary schools and surrounding communities with no previous blood surveys in northern Uganda: the prevalence after mass drug administrations and a report on suspected non-filarial endemic elephantiasis

**DOI:** 10.1186/s41182-017-0060-y

**Published:** 2017-08-15

**Authors:** Emmanuel Igwaro Odongo-Aginya, Alex Olia, Kilama Justin Luwa, Eiji Nagayasu, Anna Mary Auma, Geoffrey Egitat, Gerald Mwesigwa, Yoshitaka Ogino, Eisaku Kimura, Toshihiro Horii

**Affiliations:** 1 Department of Microbiology and Immunology, Faculty of Medicine, Gulu University, P.O. Box 166, Gulu, Uganda; 2 Department of Biology, Faculty of Science, Gulu University, P.O.Box 166, Gulu, Uganda; 3 Division of Parasitology, Department of Infectious Diseases, Faculty of Medicine, University of Miyazaki, Miyazaki, 889-1692 Japan; 4 Vector Control Division, Ministry of Health, P.O.Box 1661, Kampala, Uganda; 5 Department of Parasitology, Kochi Medical School, Kochi University, Nankoku, Kochi 783-8505 Japan; 6 Department of Haematology and Respiratory Medicine, Kochi Medical School, Kochi University, Nankoku, Kochi 783-8505 Japan; 7 Department of Molecular Protozoology, Research Institute for Microbial Diseases, Osaka University, Suita, Osaka 565-0871 Japan

**Keywords:** *Wuchereria bancrofti*, Prevalence, Mass drug administration, Podoconiosis, Uganda

## Abstract

**Background:**

A prevalence study of *Wuchereria bancrofti* infection was carried out in 2014 at 4 study sites in northern Uganda using antigen and microfilaria tests. Each study site consists of a primary school and surrounding communities. These sites are inside the filariasis endemic area and have been covered by mass drug administration under the national elimination programme. However, no prevalence study had been conducted there before the present study. Without information on past and present endemicity levels, our study was meant to be an independent third-party investigation to know the latest filariasis situation.

**Results:**

A total of 982 people including 570 schoolchildren (7–19 years) and 412 community people (7–25 years) were examined, all of them for filarial antigen and 695 for microfilariae. The study revealed that all subjects were negative by both methods.

**Conclusions:**

It was considered that annual mass drug administrations together with anti-malarial activities such as indoor residual spraying had contributed to the reduction of the filarial infection. However, based on the past data obtained near our study sites, we cannot exclude the possibility that filarial prevalence rates in our study sites were very low or even zero originally. During the study, we encountered several patients with lower leg edema and pachydermic (elephant skin-like), mossy skin lesion of the foot. Judging from clinical features and bare-footed life-style of people in the area, non-filarial elephantiasis, possibly podoconiosis, was suspected. This elephantiasis has been reported in areas where filariasis is not endemic.

## Background

Lymphatic filariasis (LF), which is one of the neglected tropical diseases (NTDs) in the world, is caused by *Wuchereria bancrofti* in Africa and transmitted mostly by *Anopheles* and *Culex* mosquitoes [1]. The adult worms parasitize the human lymphatic system and cause dilation of lymph vessels. The vessel dilation accompanied by inflammation impairs the normal drainage of lymph fluid and leads to the accumulation of the fluid or lymphedema. The edematous skin is liable to bacterial infections which can trigger an acute febrile symptom or fever attack, and repeated attacks will result in thickening of the skin and eventually disfiguring lesions known as elephantiasis [2, 3]. In the scrotum, dead adult worms may block the lymph vessel and cause accumulation of lymph fluid in the tunica vaginalis, a membranous pouch surrounding the testis. This will produce a swelling of the scrotum or hydrocele [4].

Before 2010, it was estimated that 120 million people in 81 countries were infected with LF and 40 million incapacitated or disfigured by lymphedema (including elephantiasis) and/or hydrocele [5], and World Health Organization (WHO) ranked LF as the no. 2 cause of permanent and long-term disability [6]. The Global Programme to Eliminate Lymphatic Filariasis was launched by WHO in 2000 targeting the elimination by 2020. The strategy is to give 4–6 annual mass drug administrations (MDAs) with ivermectin (or diethylcarbamazine) and albendazole covering all eligible people living in endemic areas regardless of filarial infection. An endemic area was defined as having an infection rate of ≥1% using antigen (or microfilaria) test. Approximately 1.34 billion people globally were expected to participate in the annual treatment [5].

Uganda is one of the countries in Africa, south of the Sahara, with extensive elimination activities for LF. The first epidemiological study was conducted in 1998 in Lira, Soroti, and Katakwi districts of northern and eastern Uganda. The antigen and microfilaria (*mf*) prevalence rates for the three districts were reported to be 18.3–30.1% and 9.7–25.5%, respectively. Similarly, the prevalence rates of hydrocele and limb elephantiasis in adults aged ≥20 years were 7.0–28.7% and 3.7–9.7%, respectively [7]. A country-wide distribution of LF in Uganda was subsequently assessed in Oct. 2000–Apr. 2003 with schoolchildren at 76 locations based on antigen assay. The survey showed that *W. bancrofti* infections were concentrated in the north of the Victoria Nile and the Lake Kyoga. Utilizing the same data, the first nation-wide filariasis distribution and prevalence map was prepared for the national elimination program. Further analysis, based on the map and the population (2002) data, estimated that 8.7 million people (35.3% of the national population) live in areas where antigen-positive rate of school-aged children is >1% [8].

In our study sites, which are under the national filariasis elimination program, no filariasis survey had been done before this study. A pre-treatment *mf* survey carried out in 2008 at two sites which are located relatively close to our study sites documents that no positives were found (unpublished report). An attempt to predict the pre-treatment prevalence based on published prevalence maps was not successful because the results varied so much by map. Also, we could not obtain enough information on regular annual MDAs and drug coverage and actual intake at local government offices. Under these circumstances, it was considered important to conduct an independent study, which is separate from the government initiative, to determine recent prevalence and intensity of infection. Another impetus to start this study was unconfirmed information by local medical officers that they see many cases of elephantiasis.

Meanwhile, in a study conducted in Oct./Nov. 1998 in the Mt. Elgon area of Uganda, the presence of non-filarial elephantiasis, most likely podoconiosis, was reported [9]. The disease is found in the high mountain area, where filariasis does not exist. Three cases of podoconiosis were also reported in the south-western highland in Uganda [10]. In the course of the study, we encountered several “unusual” elephantiasis cases among people walking barefoot. The findings are documented in this report.

## Methods

### Study sites

**Fig. 1:**
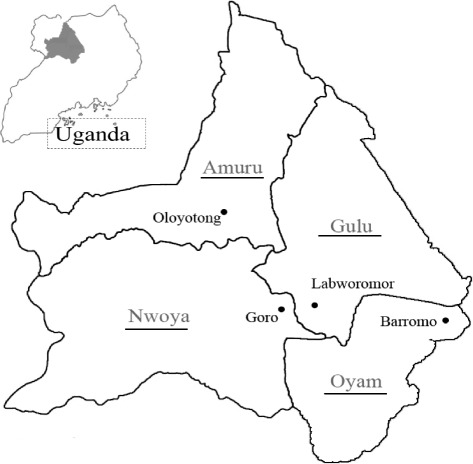
Four study sites (*bullet*), Barromo, Goro, Oloyotong, and Labworomor, each in different four districts (*underlined*). The whole Uganda map is shown in the *upper left*

The study was carried out at four study sites selected one from each of four districts of Oyam, Nwoya, Amuru, and Gulu in northern Uganda (Fig. 1). Each study site consists of a primary school and surrounding village communities. Before the site selection, the information on LF situation and elimination activities was first collected at each District Office with assistance of the District Health Officer (DHO) and District Vector Control Officer (DVCO), and one sub-county, 4–16 of which constitute one district in this study area, was selected. Then, at the sub-county office, 5–7 candidate schools were listed for blood test. All the candidate schools were visited and 4th to 7th grade schoolchildren were questioned as a class and recorded individually for their knowledge of drugs used for MDA, actual intake of the drugs and the number of clinical cases in their villages. Finally, based on summarized answers, one study school with relatively more clinical cases and lower drug intake rate was selected for the antigen and *mf* survey. Several communities surrounding the selected school were also included as part of the study site with advice from village leaders, village health team, and teachers. Hereafter, the study sites are thus designated using the names of the selected schools: Barromo, Goro, Oloyotong, and Labworomor (Fig. 1).

### Study subjects

At the selected school, male and female children of grades 1–7 were recruited. A minimum of 20 children per grade were sampled by drawing individually Yes/No lots of folded papers to get a total of >140 subjects/school. The official age to start schooling is at 6 years old, but in reality, the age per grade varies widely. In this study, children’s ages ranged from 7 to 19 years. The lowest age of 7 years was fixed by us. In the communities, >50 children aged 7–13 years, who do not go to school, and >60 people aged 14–25 years were targeted. Thus, >250 subjects/study site or >1000 subjects for the whole study were included. The subjects were first examined for antigen and only those checked for antigen were included in the *mf* study.

### Diagnostic methods

Blood collections were carried out at each of the selected school for enrolled children and a convenient point for community people. For antigen test, BinaxNow® Filariasis antigen test (AlereScarborough, Inc.) was used in the daytime following manufacturer’s instructions: 100 μl of heparinized finger-prick blood was applied to the sample pad and the result was read after 10 min. For *mf* test, approximately 60 μl of blood was collected after 8:30 pm, smeared on a glass slide which was later stained with 10% Giemsa for 10 min.

## Results

The field study was carried out in Oct.–Nov. 2014. This is in the rainy season in northern Uganda, and the work was often interrupted with strong showers. A total of 982 volunteer subjects (570 schoolchildren and 412 community people) were examined for the filarial antigen in the four study sites (Table 1A). Analyzed by age, 94.6% (*n* = 539) of schoolchildren were 7–15 years, and the rest between 16–19 years. Adults aged 20–25 years accounted for 26.5% (*n* = 109) of all community subjects. The results of the antigen test were unexpected: all of the subjects were negative.

**Table 1:** Results of *W. bancrofti* antigen (A) and microfilaria (B) tests according to study site which includes a primary school and surrounding communities

Study site	Sex	Barromo		Goro		Oloyotong		Labworomor		Total exam.
		No. exam.	No. positive	No. exam.	No. positive	No. exam.	No. positive	No. exam.	No. positive	
A										
Schoolchildren (7–19 years)	M	85	0	77	0	71	0	67	0	300
	F	55	0	64	0	76	0	75	0	270
	Total	140	0	141	0	147	0	142	0	570
Community children (7–13 years)	M	24	0	21	0	22	0	18	0	85
	F	23	0	36	0	15	0	13	0	87
	Total	47	0	57	0	37	0	31	0	172
Community people (14–25 years)	M	28	0	38	0	39	0	33	0	138
	F	28	0	22	0	22	0	33	0	102
	Total	53	0	60	0	61	0	66	0	240
Community total		100	0	117	0	98	0	97	0	412
All subjects		240	0	258	0	245	0	239	0	982
B										
Schoolchildren (7–19 years)	M	72	0	46	0	62	0	48	0	228
	F	39	0	45	0	65	0	58	0	207
	Total	111	0	91	0	127	0	106	0	435
Community children (7–13 years)	M	19	0	12	0	19	0	16	0	66
	F	17	0	23	0	12	0	10	0	62
	Total	36	0	35	0	31	0	26	0	128
Communitypeople (14–25yrs)	M	20	0	29	0	19	0	10	0	78
	F	15	0	13	0	13	0	13	0	54
	Total	35	0	42	0	32	0	23	0	132
Community total		71	0	77	0	63	0	49	0	260
All subjects		182	0	168	0	190	0	155	0	695

In community surveys by the antigen and microfilaria tests, schoolchildren are excluded

For *mf* test, a total of 695 subjects, which is 70.8% of those tested for antigen, were examined at night from 8:30 pm to midnight (Table 1B). Schoolchildren and community children aged 7–13 years participated in the *mf* survey at the rates of 76.3 and 74.4%, respectively, but for community youths and adults (14–25 years), participation was only 55.0% of the total tested for antigen. Only 29 adults (20–25 years) were examined for *mf*. All slides examined were negative for *W. bancrofti mf*, and no *mf* of another species, *Mansonella perstants*, which is distributed in Uganda [11], were found in any of the blood smears either.

**Fig. 2:**
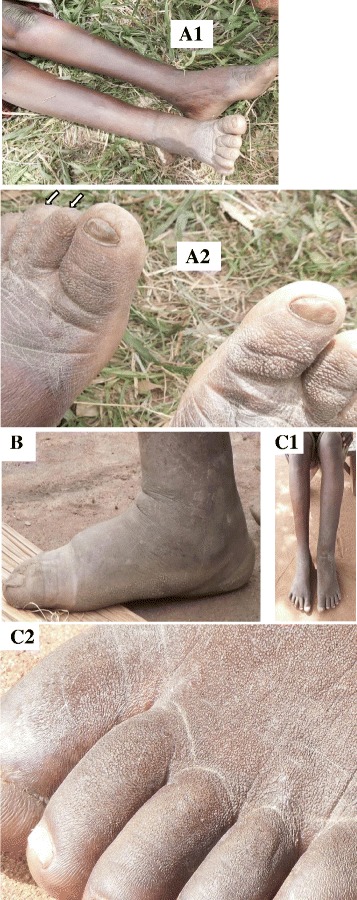
Three cases (**a**, **b**, and **c**) with leg edema and pachydermic skin changes. In case **a**, the *right lower* leg is slightly edematous and the dorsal skin of the *right* foot is whitish in appearance (**a1**). The skin of the foot is pachydermic and mossy: the skins of the *right* toes are rather spiny, and the nails of the *left* 2nd and 3rd toes are unrecognizable (*arrows*) (**a2**). In case **b**, foot edema and pachydermic skin change along the side of the foot are recognized (**b**). Mild edema is observable in the *left lower* leg of case **c** (**c1**), and the skin around the *left* toes is pachydermic (**c2**)

During the blood surveys, about 7–8 male and female adults visited us asking advice for their “filarial” elephantiasis. The lesions were basically similar: lower leg edema, and fine pachydermic (which is like a rough elephant skin) or mossy skin change on the feet. The photographs of three cases (a, b, and c) are shown in Fig. 2. The edema is slight or moderate and confined below the knee (Fig. 2a1–c1). The skin lesion, which is typically bilateral and severer at one side, is most prominent around toes (Fig. 2a2–c2). In case a, some toe nails are unrecognizable (Fig. 2a2, arrows). In case b, a pachydermic lesion is observed along the side of the foot (Fig. 2b). Most people in these areas walk and work barefooted. Based on this life style, clinical picture and negative antigen/*mf* results (with the assumption that our sites are non-endemic originally), we conclude that a possibility of non-filarial endemic elephantiasis, podoconiosis, should be considered.

## Discussion

A total of 982 people were examined with the antigen test, and 695 of them (70.8%) were also examined for *mf*, despite the fact that the second blood collection was done at night with unpredictable downpours. The results of the blood tests turned out to be all negative. This can be explained, in general, by the effect of anti-filarial MDAs. They have been implemented in Oyam district since 2005 when the district was a part of Apac district and in the other three districts since 2009. Before our study in 2014, 8, 4, and 3 rounds of annual MDAs were conducted by the national program in Oyam, Amuru and Gulu, and Nwoya districts, respectively. The treatment coverage was satisfactory (>65%) in Oyam, Amuru and Nwoya, but in Gulu three of four rounds resulted in low coverage rates of 34–58%. However, specific rates for our study sites are unknown. Various anti-malarial activities might have also influenced the result, though quantitative effects of these interventions to reduce the prevalence of filarial infection are unknown. Between 2009 and 2014, our four districts were covered by the intensive indoor residual spraying (IRS) program for malaria control [12]. It is reported that five rounds of IRS, especially using bendiocarb, reduced malaria morbidity significantly in Apac district [13], implying that IRS would reduce FL transmission as the two different parasites share the same vector species of *Anopheles*. A mass distribution campaign of long-lasting insecticidal nets (LLINs) initially focused on pregnant women and children under 5 years in 2009–2010 and then expanded in 2013–2014 to nationwide universal distribution of LLINs. In Mid-North region including our study districts, each household was reported to own an average of 2.7 LLINs [12, 14]. The possible supportive effect of LLINs in anti-filarial MDAs is reported [15].

**Fig. 3:**
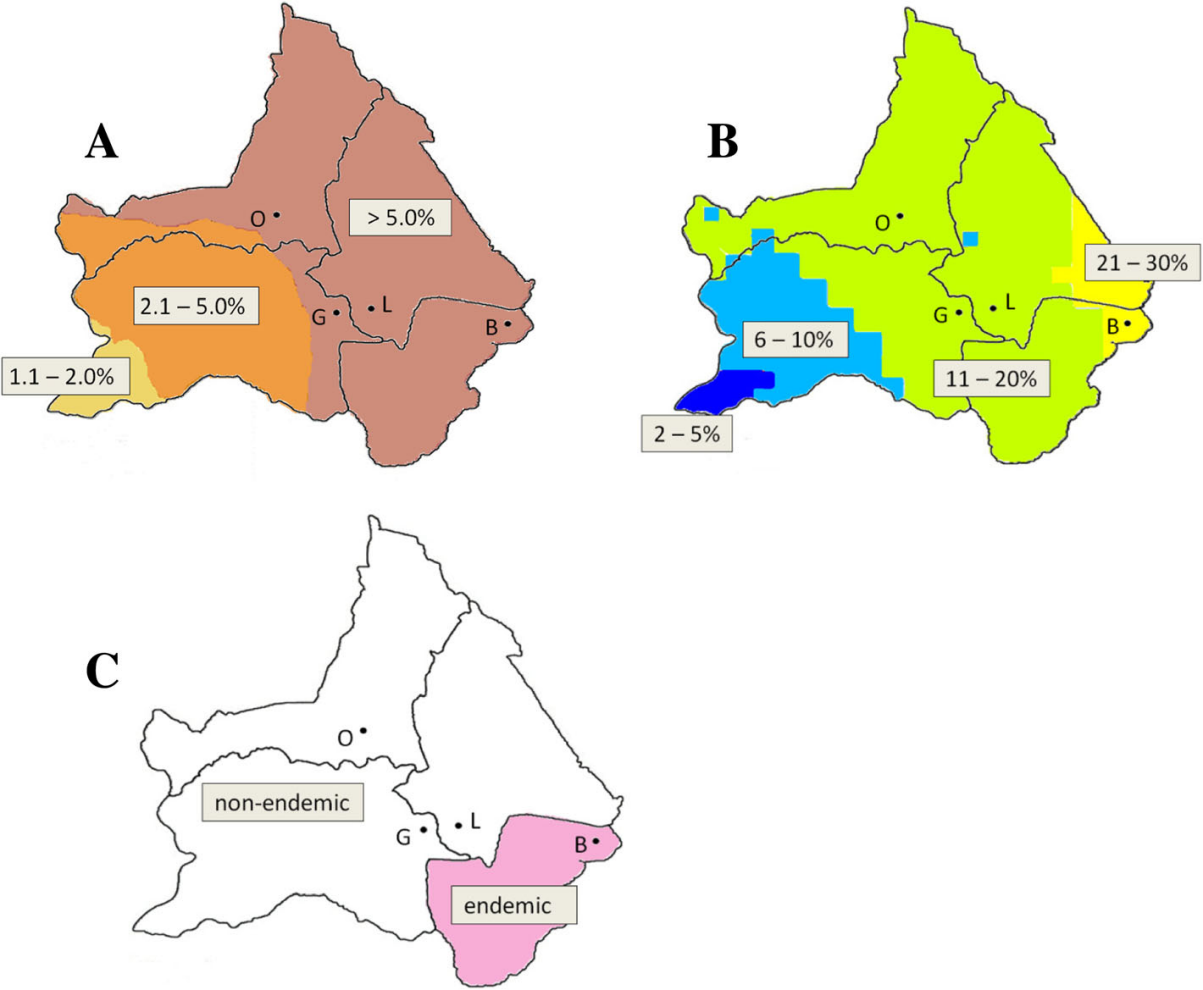
Three published maps showing prevalence or endemicity of bancroftian filariasis in Uganda. Our four study sites are plotted on each map. *O* Oloyotong, *G* Goro, *L* Labworomor, *B* Barromo. **a** The map reported by Onapa et al. [8]. All of our four study sites are in the >5.0% endemic zone. **b** The map is from Global Atlas of Helminth Infections (GAHI). The original map is reported by Moraga et al. [16]. Three of our sites are in the 11–20% prevalence zone, and one in the 21–30% zone. **c** The map is reported by Kolaczinski et al. [17]. Three of our sites are in the districts classified as non-endemic, and one in the endemic district

To understand the totally negative results, crucial information is the pre-MDA infection level in our study sites. In order to have rough estimates of the pre-MDA rates, the geographical positioning system (GPS) data of our study sites (recorded at the schools) were first plotted on the antigen prevalence map made by Onapa et al. [8], which gave a rate of >5% at all four sites (Fig. 3a). Since the map by Onapa et al. was created based on a limited number of samples (*n* = 76) scattered across the whole country, the prevalence map could not be accurate for our purpose. However, based on the predicted estimate (>5%), we still anticipated some positives in our study even after treatment. As for the comparability between predicted rates from the map and antigen rates by our study, the average number of subjects examined and the age range in Onapa et al. [8] are 231 persons/school and 5–19 years, and ours are 218 persons/site and 7–19 years excluding adults aged 20–25 years. Interestingly, by the use of a newly published (2015) pre-MDA antigen prevalence map for Uganda [16], we predicted high rates of 21–30% in Barromo and 11–20% in the other three sites (Fig. 3b). Surprisingly, another filariasis distribution map of Uganda published in 2007 for the integrated control of NTDs [17] excludes three of our districts, Nwoya, Amuru, and Gulu from the LF endemic area (Fig. 3c). In fact, in the pre-treatment *mf* survey carried out in July–October 2008 by the national elimination program, no positives were recorded for two sites, Koch Goma in Nwoya district (*n* = 442) and Opit in Gulu district (*n* = 354) [previously mentioned unpublished report]. The former site is located 10 km from our Goro site and the latter 29 km from our Labworomor site. Then it could be inferred, based on the relative proximity, that our two sites had very low infection rates when MDA started or that the sites are non-endemic from the beginning. This conclusion, if confirmed, will be a challenge to the reliability of computer-based mapping. Barromo site belongs to a more endemic region, and its pre-treatment prevalence might be higher than those of the other three sites. Prolonged and more intensive MDAs could explain the zero prevalence we obtained there.

During this study, we encountered patients with unique elephantiasis: the leg edema is mild and restricted below the knee, but mossy skin change around their toes is obvious. We also noticed that most people walk and work barefooted. Thus, non-filarial elephantiasis such as podoconiosis was suspected. Podoconiosis is a well-known non-filarial endemic elephantiasis found in high mountain areas (1000–2800 m) in Ethiopia, where weathered volcanic ash containing silicon predominates in soil and people live barefoot in a cool and relatively rainy (annual precipitation of >1000 mm) environment [18]. A pathological study revealed amorphous and crystalline silicon compounds in macrophages of femoral lymph nodes [19]. In Uganda, podoconiosis cases were reported from highlands of >1500 m [9, 10], while LF distribution was limited in areas lower than 1300 m [8]. In Ethiopia, however, potential distribution overlap of these two different types of elephantiasis is reported at an altitude of 1225−1698 m [20], and one report clearly mentions the co-endemicity in western Ethiopia [21]. Our study sites are located at 1086–1119 m, which is lower than the reported endemic range. However, a recent report that quartz, crystallized silicon dioxide, is closely related to the occurrence of podoconiosis [22] may suggest wider distribution of podoconiosis because quartz is ubiquitous in the environment, and its microscopic particles may be absorbed through the foot skin. Another supportive reason to suspect podoconiosis is found in the previously mentioned unpublished report from Koch Goma and Opit of 931 people examined clinically, 13 elephantiasis (1.40%), and 1 hydrocele (0.11%) cases were found (*p* < 0.002). It is quite unusual to have a significantly higher elephantiasis rate than a hydrocele rate in LF endemic areas [23, 24]. In 2011, WHO identified podoconiosis as one of NTDs and its elimination has been considered [25], and it will be important for us to confirm the diagnosis, though the definite diagnosis of podoconiosis is not easy [26]. If we can confirm the absence of LF in the area, it can be a strong supportive evidence for podoconiosis, and for this, *W. bancrofti* antibody test may be valuable.

Although silicon compounds play an essential role, the exact pathogenesis of podoconiosis is yet unknown. The skin lesions we observed are mild compared with those reported in various articles [27–29]. It may be possible that we encountered early stage cases. It is also possible that the skin lesion is influenced by the difference in the concentration and constitution of silicon compounds in soil. The severity may also be influenced by genetic difference between people/population [30].

## Conclusions

Two of the computer-based maps predicted relatively high prevalence of LF infections in our study sites, but no antigen/*mf* positives were detected by our study. Also, in another study carried out in nearby areas before MDAs, no *mf* positives were found. Thus, we suspected the possibility that our area was originally non-endemic (or very low endemic). The present study also suggests a possibility that non-filarial elephantiasis or probable podoconiosis could be co-endemic with filarial elephantiasis in Uganda. It is necessary to confirm the diagnosis and investigate the clinical and epidemiological significance of “unusual” elephantiasis in Uganda.

## Abbreviations

IRS: Indoor residual spraying
LF: Lymphatic filariasis
LLINs: Long-lasting insecticidal nets
MDAs: Mass drug administrations
*Mf*: Microfilaria
NTDs: Neglected tropical diseases
WHO: World Health Organization
