# Erratum to: *Wuchereria bancrofti* infection at four primary schools and surrounding communities with no previous blood surveys in northern Uganda: the prevalence after mass drug administrations and a report on suspected non-filarial endemic elephantiasis

**DOI:** 10.1186/s41182-017-0068-3

**Published:** 2017-09-20

**Authors:** Emmanuel Igwaro Odongo-Aginya, Alex Olia, Kilama Justin Luwa, Eiji Nagayasu, Anna Mary Auma, Geoffrey Egitat, Gerald Mwesigwa, Yoshitaka Ogino, Eisaku Kimura, Toshihiro Horii

**Affiliations:** 1 Department of Microbiology and Immunology, Faculty of Medicine, Gulu University, P.O. Box 166, Gulu, Uganda; 2 Department of Biology, Faculty of Science, Gulu University, P.O.Box 166, Gulu, Uganda; 3 Division of Parasitology, Department of Infectious Diseases, Faculty of Medicine, University of Miyazaki, Miyazaki, 889-1692 Japan; 4 Vector Control Division, Ministry of Health, P.O.Box 1661, Kampala, Uganda; 5 Department of Parasitology, Kochi Medical School, Kochi University, Nankoku, Kochi 783-8505 Japan; 6 Department of Haematology and Respiratory Medicine, Kochi Medical School, Kochi University, Nankoku, Kochi 783-8505 Japan; 7 Department of Molecular Protozoology, Research Institute for Microbial Diseases, Osaka University, Suita, Osaka, 565-0871 Japan

## Erratum

In the original publication of this article [1] there was 1 incorrect value displayed in Table 1. In this Erratum the incorrect (Table 1 in this Erratum) and correct version of Table 1 are shown (Table 2 in this Erratum). Only a part of Table 1 is shown in this Erratum. The full version of Table 1 is available in the original article. The original publication has been updated with the correct data; the error did not impact the scientific outcome of the study. The publisher apologizes to the authors and readers for the inconvenience.

**Table 1:** Original version of Table 1 as published on 15 August 2017

Study site	Sex	Barromo	
		No. exam.	No. positive
Community people (14–25 years)	M	28	0
	F	28	0
	Total	53	0

**Table 2:** Correct version of Table 1, the value 28 has been corrected to 25

Study site	Sex	Barromo	
		No. exam.	No. positive
Community people (14–25 years)	M	28	0
	F	25	0
	Total	53	0
